# Micropatterned composite membrane guides oriented cell growth and vascularization for accelerating wound healing

**DOI:** 10.1093/rb/rbac108

**Published:** 2022-12-26

**Authors:** Jiaqi Li, Xulong Liu, Weiyong Tao, Yan Li, Yingying Du, Shengmin Zhang

**Affiliations:** Advanced Biomaterials and Tissue Engineering Center, Huazhong University of Science and Technology, Wuhan 430074, China; NMPA Research Base of Regulatory Science for Medical Devices & Institute of Regulatory Science for Medical Devices, Huazhong University of Science and Technology, Wuhan 430074, China; Institute of Biomaterials and Medical Devices, Wuhan Institute of Industrial Innovation and Development, Wuhan 430074, China; Department of Biomedical Engineering, Huazhong University of Science and Technology, Wuhan 430074, China; Advanced Biomaterials and Tissue Engineering Center, Huazhong University of Science and Technology, Wuhan 430074, China; NMPA Research Base of Regulatory Science for Medical Devices & Institute of Regulatory Science for Medical Devices, Huazhong University of Science and Technology, Wuhan 430074, China; Institute of Biomaterials and Medical Devices, Wuhan Institute of Industrial Innovation and Development, Wuhan 430074, China; Department of Biomedical Engineering, Huazhong University of Science and Technology, Wuhan 430074, China; Advanced Biomaterials and Tissue Engineering Center, Huazhong University of Science and Technology, Wuhan 430074, China; NMPA Research Base of Regulatory Science for Medical Devices & Institute of Regulatory Science for Medical Devices, Huazhong University of Science and Technology, Wuhan 430074, China; Institute of Biomaterials and Medical Devices, Wuhan Institute of Industrial Innovation and Development, Wuhan 430074, China; Department of Biomedical Engineering, Huazhong University of Science and Technology, Wuhan 430074, China; Advanced Biomaterials and Tissue Engineering Center, Huazhong University of Science and Technology, Wuhan 430074, China; NMPA Research Base of Regulatory Science for Medical Devices & Institute of Regulatory Science for Medical Devices, Huazhong University of Science and Technology, Wuhan 430074, China; Institute of Biomaterials and Medical Devices, Wuhan Institute of Industrial Innovation and Development, Wuhan 430074, China; Department of Biomedical Engineering, Huazhong University of Science and Technology, Wuhan 430074, China; Advanced Biomaterials and Tissue Engineering Center, Huazhong University of Science and Technology, Wuhan 430074, China; NMPA Research Base of Regulatory Science for Medical Devices & Institute of Regulatory Science for Medical Devices, Huazhong University of Science and Technology, Wuhan 430074, China; Institute of Biomaterials and Medical Devices, Wuhan Institute of Industrial Innovation and Development, Wuhan 430074, China; Department of Biomedical Engineering, Huazhong University of Science and Technology, Wuhan 430074, China; Advanced Biomaterials and Tissue Engineering Center, Huazhong University of Science and Technology, Wuhan 430074, China; NMPA Research Base of Regulatory Science for Medical Devices & Institute of Regulatory Science for Medical Devices, Huazhong University of Science and Technology, Wuhan 430074, China; Institute of Biomaterials and Medical Devices, Wuhan Institute of Industrial Innovation and Development, Wuhan 430074, China; Department of Biomedical Engineering, Huazhong University of Science and Technology, Wuhan 430074, China

**Keywords:** topology, cell behavior, wound healing, gelatin, polymer biomaterials

## Abstract

Skin defect is common in daily life, but repairing large skin defects remains a challenge. Using biomaterials to deliver biochemical or physical factors to promote skin tissue regeneration is of great significance for accelerating wound healing. Specific surface micropatterns on biomaterials could affect cell behavior and tissue regeneration. However, few studies have focused on the construction of wound healing biomaterials with surface micropatterns and their role in skin tissue regeneration. In the present study, gelatin–polycaprolactone/silk fibroin composite membranes with different micropatterns were fabricated by photolithography, including line, grid and plane micropatterns. *In vitro* cell experiments demonstrated that the line micropattern on the composite membrane could guide cell-oriented growth, and more importantly, promote the expression of angiogenesis-related markers and α-smooth muscle actin (α-SMA) at both gene level and protein level. In the rat full-thickness skin defect model, the composite membrane with line micropatterns increased α-SMA production and neovascularization in wounds, leading to accelerated wound contraction and healing. The current study not only suggests that composite membranes with specific micropatterns can be promising wound repair materials but also provides new insights into the importance of biomaterial surface topology for tissue regeneration.

## Graphical abstract

**Figure:**
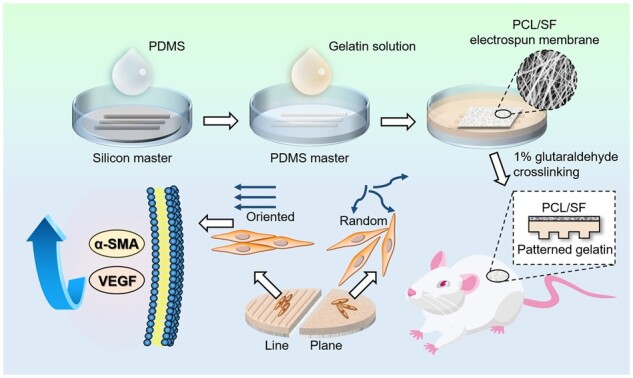

## Introduction

Skin is the largest organ in the human body and plays an important role in isolating human tissues from the external environment [1]. In addition to the barrier function, skin also provides a critical role in secretion, touch, respiration, metabolism, etc. [2]. Skin wounds caused by acute trauma or chronic diseases are very common in life. Inadequate wound healing often occurs in severe skin damage such as large-scale thermal injuries, followed by a loss of skin organ function rendering the organism vulnerable to infections, thermal dysregulation and fluid loss [3]. Conventional therapies such as autologous or allogeneic skin grafts can re-epithelialize mid-thickness wounds [4]; however, the source of autologous skin is limited, and allogeneic skin may cause severe immune rejection, which greatly reduces their practicability in the treatment of large wounds. Therefore, suitable artificial skin materials have great clinical value.

The ideal biomaterials for wound healing should be biocompatible, soft, antibacterial and provide a moist environment to promote wound healing [5]. Furthermore, using biomaterials to deliver biochemical or physical factors to promote skin tissue regeneration is of great significance for accelerating wound healing. Previous studies have suggested that providing a microenvironment with specific topological structures could significantly influence cell fate [6]. Surface topology of materials also transmit signals to cells [7, 8], affecting cell behavior, including adhesion, proliferation, migration, differentiation, etc. [9, 10]. For example, the construction of micropatterns on the surface of biomaterials has become an effective strategy to regulate cell fate and promote tissue repair, including bone, periosteum and so on [11]. In the wound healing field, studies have shown that the directional proliferation of cells can promote the shrinkage and healing of skin wounds [12–14], inspired by the orientated arrangement of cells in natural skin. However, few studies have focused on the construction of wound healing biomaterials with surface micropatterns and their role in skin tissue regeneration.

In this study, we constructed a micropatterned composite membrane that mimics the fibrous structure of skin to promote wound healing. Briefly, the bi-layered composite membrane was composed of a striped patterned gelatin membrane and a polycaprolactone (PCL)/silk fibroin (SF) electrospun membrane. The patterned gelatin side that mimics the fibrous structure of natural skin could guide cell-oriented growth. The PCL/SF electrospun membrane was designed to improve the mechanical properties and continued to protect the wound after gelatin was degraded. The *in vitro* cell experiments showed that the composite membrane could guide the oriented growth of cells, and promote the secretion of vascular endothelial growth factor a (VEGFA) and α-smooth muscle actin (α-SMA). A rat full-thickness skin defect model experiment demonstrated that the composite membrane with line micropatterns increased α-SMA production and neovascularization in wounds, leading to accelerated wound contraction and healing.

## Materials and methods

### Materials and reagents

Silicon wafer with crystal phase of 100 was purchased from RDMICRO (Suzhou, China). Concentrated sulfuric acid, hydrogen peroxide, glutaraldehyde and PCL (80 000 Mw) were obtained from Chinese Medicine Group. SU-8 GM 1070 photoresist were from Gersteltec Sarl, CH. Polydimethylsiloxane (PDMS) was from Dow Corning Corp, USA. Diacetone alcohol and Propylene Glycol Monomethyl Ether Acetate (PGMEA) were purchased from Micro-Chem, USA. 1,1,1,3,3,3-Hexafluoroisopropanol (HFIP) was purchased from Sigma-Aldrich, USA. RPMI Medium 1640 basic (Gibco, USA), low glucose DMEM Medium (Gibco, USA), fetal bovine serum (HyClone, USA), penicillin–streptomycin solution (HyClone, USA), trypsin (HyClone, USA) and PBS (HyClone, USA) were used for cell culture. NCTC clone 929 (L-929) and bone mesenchymal stem cells (MSCs) were purchased from Cyagen.

### Fabrication of patterned gelatin–PCL/SF composite membranes

#### Fabrication of PDMS stamp

Micro patterns with different sizes and different shapes (line pattern and grid pattern) were prepared on photomask by high-resolution laser printing. Silicon wafers were first cleaned with piranha solution (4:1 mixture of concentrated sulfuric acid and hydrogen peroxide) and treated by oxygen plasma for 2 min. Through standard photolithography technology, silicon masters with these designed patterns were produced by SU-8 GM 1070 on silicon wafers. Silicon master was placed in a plastic dish and covered by debubbled PDMS pre-polymer on a horizontal surface. After cured at 60°C in the oven overnight, the PDMS stamp was torn from the silicon master.

#### Fabrication of PCL/SF electrospinning membranes

SF was prepared using the protocol we have reported [15]. PCL and SF were prepared in a light-proof brown bottle at a mass ratio of 4:1, and HFIP was added to make a solution of 15 wt%, then stirred until PCL and SF were completely dissolved; 2.0 ml of electrospinning solution was fed at speed of 0.04 ml/min, and the electrospinning membrane was produced with the drum speed of 50 rpm, high voltage of 15 kV and separation distance of 10 cm.

#### Fabrication of composite membranes

**Scheme 1.**
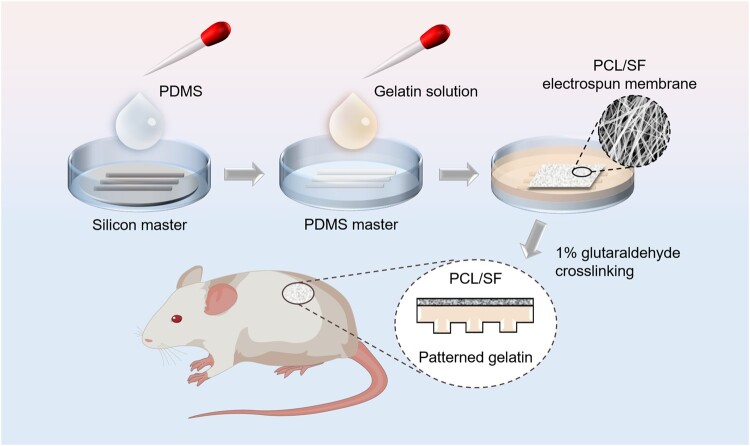
Procedure of patterned gelatin–PCL/SF composite membrane for repairing full-thickness skin defects in rats.

The bottom of the PDMS stamp was fully attached to the glass dish by oxygen plasma treatment for 2 min, then cured in a 95°C oven for 15 min to make the bond permanent. A 10 wt% gelatin solution was prepared with ultrapure water and heated in a water bath until completely dissolved; 15 ml gelatin solution was added into the PDMS stamp-bonded glass dish, and after removal of air bubbles, gelatin was solidified at 4°C for 30 min. The surface of the gelatin gel was covered with the PCL/SF membrane, and the gelatin penetrated into the gap of the fibers to make them tightly bounded. After cured at 35°C for 5 h, the gelatin–PCL/SF composite membrane was cross-linked by 1% glutaraldehyde solution for 20 min. The composite membrane was soaked in 1M glycine aqueous solution and shaken for 3 days. The glycine solution was replaced every 12 h until the solution was transparent and colorless, and then the composite membrane was cleaned in ultrapure water for 1 day to remove residual glutaraldehyde. The flowchart of key steps is shown in Scheme 1.

### Characterization of composite membranes

Topographical features of the composite membrane were examined by scanning electron microscope (SEM) (Nova NanoSEM 450, FEI, Netherlands) and inverted fluorescence microscope (Ti-S, Nikon, Japan). The surface topography of the electrospun fibers and the longitudinal cross-sectional topography of the composite membrane were observed by SEM. The micropatterns on the surface of the gelatin membrane were observed by inverted fluorescence microscope. The depths of the patterns in the gelatin membranes were measured using an ultra-depth-of-field microscope (VHX-7000, KEYENCE, Japan). The composite membranes were cut into strips of 1 × 4 cm, and stretched to break at a rate of 5 mm/min with microcomputer controlled electronic universal testing machine (CMT4104, MTS Systems (China) Co., Ltd.). The thickness of each composite membrane was measured using a vernier calliper. The stretch direction was parallel to the line patterned composite membranes. With 10 samples per experimental group, the elastic modulus and tensile strength of the composite membranes were calculated, and the stress–strain curves were generated.

### Cell orientation on patterned membranes

MSCs and L-929 cells were cultured in petri dishes with low glucose DMEM and RPMI 1640 medium containing 10% fetal bovine serum and 1% penicillin–streptomycin solution, and kept in humidified incubator (HS40, Thermo Scientific, USA) at 37°C with 5% CO_2_.

Cells were seeded on the composite membranes with line patterns, grid patterns and plane patterns on the surface after filled the bottom of the petri dish. The composite membranes were soaked in the medium for 2 h before seeding cells to replace the water in gelatin gel. After cultured for 2 days, cells were fixed with 4% paraformaldehyde and stained with DAPI (Cyagen, USA) and acti-stain^TM^ 488 phalloidin (Cytoskeleton, USA). The cell morphology was observed through confocal laser scanning microscope (FV3000, Olympus, Japan). Cell orientation, defined as the angle between the cell and patterns, was measured by Image J software. The lateral direction was denoted as 0°.

### Cell proliferation

The experimental groups were line pattern composite membrane group, grid pattern composite membrane group and no-patterned composite membrane group. There were five samples at each time point in each group, and the mean and standard deviation (SD) were calculated. The L-929 cells in petri dish were digested and centrifuged, and 1.2 × 10^3^ cell suspension per well was added to each experimental group in a 24-well plate, while MSCs were seeded at a density of 8 × 10^3^ cells per well. Cells were cultured in a carbon dioxide incubator for 7 days with medium changes every 48 h, then treated with CCK-8 working solution at the time points of days 1, 3, 5 and 7. Absorbance values were measured using a microplate reader (Elx808, Biotek, USA).

### Quantitative real-time PCR for cells cultured on patterns

MSCs and L-929 cells were seeded on line patterns, grid patterns and plane patterns. After 24 and 72 h in culture, total RNA was harvested using SteadyPure Universal RNA Extraction Kit (Accurate Biology, China). Complementary DNA was synthesized from 500 ng of total RNA using Evo M-MLV RT Premix for qPCR Kit (Accurate Biology, China). Sequences of primers used for gene expression analysis are listed in Supplementary Table S1. Specific primers were designed using Primer-BLAST on the National Centre for Biotechnology Information website. The mRNA expression of VEGFA, α-SMA, angiopoietin1 (Angpt1) and angiopoietin2 (Angpt2) was assayed on QS3 (Applied Bio-systems, Foster City, CA, USA) using SYBR Green Premix Pro Taq HS qPCR Kit (Accurate Biology, China) reagent. Relative gene expression was normalized against the house keeping gene GAPDH and calculated by 2^–ΔΔCt^ method.

### SDS and western blotting for cells cultured on patterns

MSCs and L-929 cells were seeded on line patterns, grid patterns and plane patterns. Whole cell lysates were harvested from cells after 24 and 72 h in culture. Cell lysates were quantified by bicinchoninic acid assay (Thermo, USA); 20 µg of cell lysates were loaded with 10 µl of loading buffer and heated at 95°C for 20 min. Both samples were separated by 10% SDS–polyacrylamide gel in Mini-Protean TGX electrophoresis apparatus (Bio-Rad, USA). The proteins were run in the stacking gel at 80 V for 20 min. After the proteins passed the stacking gel and entered the resolving gel, the voltage was increased to 120 V for 60 min. At the time of running, the proteins, polyvinylidene fluoride (PVDF) membrane was soaked into the 100% methanol to remove its hydrophobicity character. After the completion of SDS electrophoresis, the gel was stacked between the PVDF membrane and blotting paper and run in transfer buffer at 200 mA for 2 h to transfer the proteins from the gel to the PVDF membrane (0.22 μm Millipore, Bedford, MA, USA). The membranes were incubated in blocking buffer (5% skimmed milk in tris buffer with Tween 20) at room temperature for 1 h before incubated overnight at 4°C with several primary antibodies, including anti-VEGFA (1:1000, mouse monoclonal, Proteintech, China), anti-α-SMA (1:1000, rabbit polyclonal, Proteintech, China) and anti-GAPDH (1:50 000, mouse monoclonal, Proteintech, China). After washing, the membranes were incubated with anti-rabbit Dylight 680-conjugated secondary antibody and anti-mouse Dylight 680-conjugated secondary antibody (Rockland, USA) at a dilution of 1:5000. Protein bands were visualized using the Odyssey infrared imaging system (LI-COR Biosciences, Lincoln, NE, USA) and densitometry was performed using Image J software (National Institutes of Health, Bethesda, MD, USA).

### *In vivo* wound healing evaluation

All procedures of *in vivo* experiment were performed in accordance with regulations approved by the Institutional Animal Care and Use Committee of Huazhong University of Science and Technology. Male Sprague-Dawley rats (hlkbio, Hubei, China) weighing 280–320 g were used to establish full-thickness skin defect model. Four full-thickness skin wounds with a diameter of 13 mm were created on the back of the rat using surgical scissors after depilation of the dorsal skin. The wounds were covered with line-patterned composite membrane, grid-patterned composite membrane, no-patterned composite membrane and paraffin gauze as a control, and were sutured with surgical sutures. The thickness of each composite membrane was ∼0.8 mm. At Days 3, 7 and 14, the wounds were photographed with a camera, and the actual areas of the healing wounds were measured by Photoshop software. The percentage of relative wound area was calculated as: area of actual wound/area of original wound × 100%.

### Histological analysis

At each time point (Days 3, 7 and 14), five randomly selected rats were sacrificed by an overdose of sodium pentobarbital. The wound tissue was cut out and fixed by 4% paraformaldehyde overnight, and then embedded in paraffin. After cryosectioning, the samples were proceeded with hematoxylin and eosin (H&E) and Masson’s trichrome staining; 10% donkey serum was used for blocking nonspecific bindings. For IF double-staining, the sections were incubated with two kinds of primary antibodies, mouse anti-CD31 (Abcam, USA) and rabbit anti-α-SMA (Abcam, USA), followed by staining with fluorescently conjugated secondary antibodies, donkey anti-mouse Alexa Fluor^®^ 488 and donkey anti-rabbit Alexa Fluor^®^ 555. Stained sections were observed by confocal microscopy imaging, and Image J was used for semi-quantitative assessment of angiogenesis and α-SMA expression. Ratio of α-SMA fluorescence intensities was calculated through ‘Mean gray value’ with selecting ‘Limit to threshold’. Anti-CD31 stained the vascular wall and formed a closed ring. The sum number of rings was counted to get the amount of vessels. The ratio between the total sectional area of blood vessels and the area of visual field was calculated to get ratio of vascular area.

### Statistical analysis

All quantitative data were expressed as mean ± SD. Statistical differences were analyzed by one-way analysis of variance.

## Results

### Characterization of gelatin–PCL/SF composite membranes

**Figure 1.**
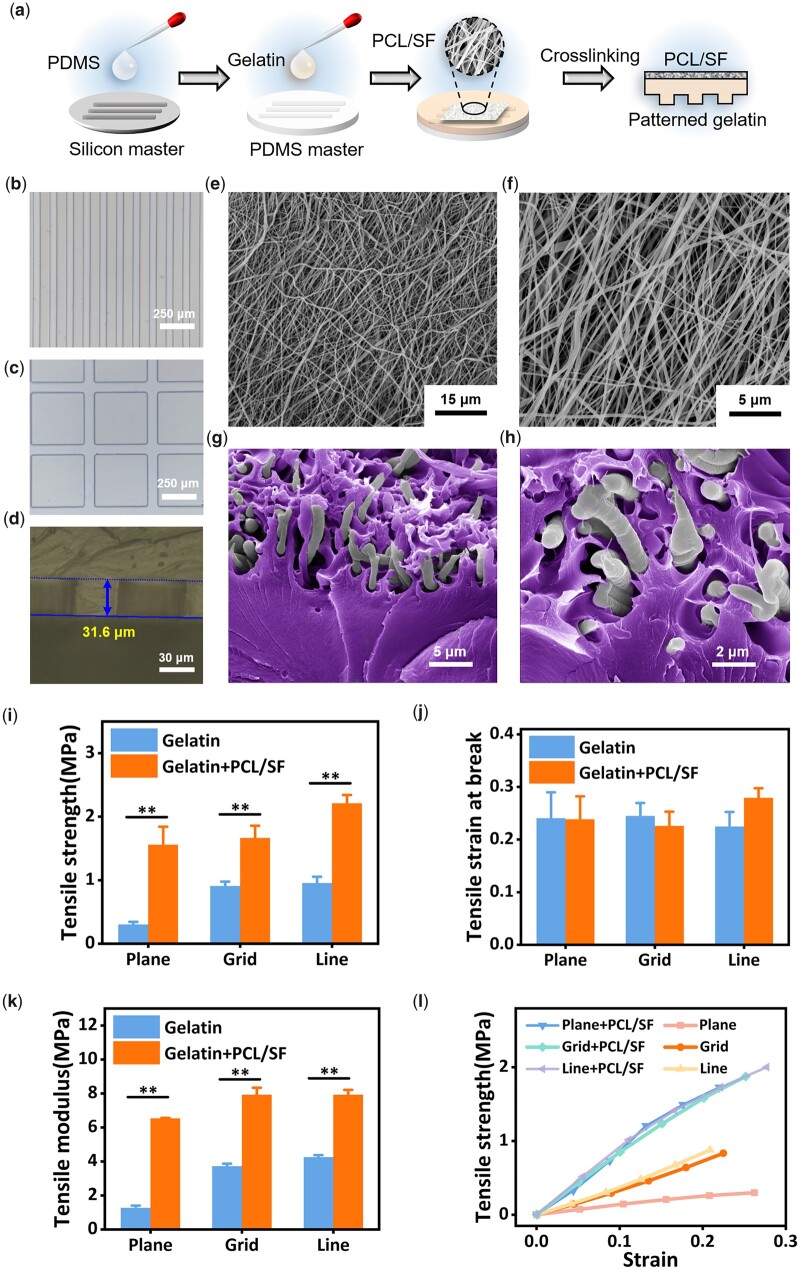
(**a**) Preparing process of patterned gelatin–PCL/SF composite membranes; (**b** and **c**) microscope images of gelatin membranes with different patterns; (**d**) depth of the patterns on gelatin membranes; (**e** and **f**) SEM images of PCL/SF electrospun fibers; (**g** and **h**) pseudo color SEM images of cross sections in composite membrane. Gelatin was marked as purple. (**i**–**k**) Tensile strength, tensile strain at break and tensile modulus of patterned gelatin and gelatin–PCL/SF composite membranes. **P < 0.01; (**l**) Tensile strength–strain curve of patterned gelatin–PCL/SF composite membranes and gelatin membranes.

The preparation procedures of the patterned composite membranes were listed in Section ‘Fabrication of patterned gelatin–PCL/SF composite membranes’ (Fig. 1A). As shown in Fig. 1B and C, the gelatin membrane obtained by PDMS master matched the pattern designed by CAD software, with relatively high accuracy and sharp edges. However, due to the swelling property of gelatin, it would swell to a certain extent after absorbing water, and the patterned gelatin membrane slightly deformed compared with the CAD design. The convex part of the gelatin membrane expanded by ∼8 μm, and the concave part shrunk by ∼8 μm. This variation was negligible in larger-sized patterns.

The depth of the pattern was measured by ultra-depth-of-field microscope (Fig. 1D). The pattern depth on the surface of the gelatin layer of the composite membrane ranged from 30 to 35 μm, which equaled with the height of the pattern on the silicon master. In the photolithography process, the factors affecting the pattern height included the amount of photoresist used, the rotational speed, the size of the pattern, the precision of the photolithography machine, etc. It is shown that the electrospun fibers prepared from PCL and SF were relatively uniform, with fiber diameters around 1 μm (Fig. 1E and F). After being covered with the PCL/SF electrospun membrane, the low-temperature gelatin gel penetrated into the gap of the electrospun fibers (Fig. 1G and H), which formed a sturdy composite membrane after drying and cross-linking. Compared with the pure gelatin membrane, the composite membrane had no obvious change in the deformation caused by tensile fracture, while the tensile strength and tensile modulus were significantly improved (Fig. 1I–K). Through tensile stress–strain curves (Fig. 1L), composite membrane also performed better with relatively higher tensile modulus.

### Cell culture on composite membrane

**Figure 2.**
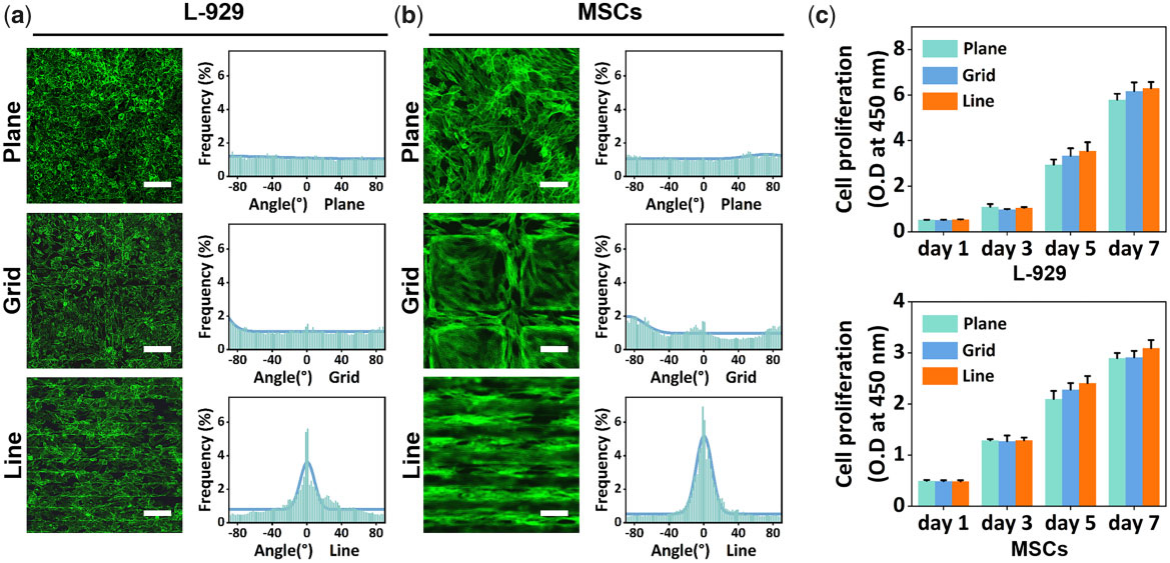
(**a** and **b**) Confocal images of F-actin from L-929 cells and MSCs on patterned gelatin membranes (scale bars: 120 μm); (**c**) proliferation of L-929 cells and MSCs grown on patterned gelatin membranes.

L-929 cells and MSCs were cultured on different patterns of gelatin membranes for 48 h. Using laser confocal microscope, we were able to observe the stained F-actin of cells and analyze the stretching direction of cells. To explore the optimal intervals between the line pattern to guide the oriented growth of cells, L-929 cells were cultured on line-patterned gelatin membranes with different intervals: 30; 50; 70 and 100 μm. As shown in Supplementary Fig. S1, the groups with 30 and 50 μm intervals presented higher cell orientation than the other two groups. To avoid the possibility of poor cell growth in the narrow concave portion, line pattern with a spacing of 50 μm was selected in the follow-up experiments. The angles between the F-actin of cells and the patterns were measured using directionality tool of Image J software. As shown in Fig. 2A and B, compared with the grid pattern and plane control, the line pattern group showed significantly higher cell orientation. A larger proportion of cells grew along the direction of the line pattern, while randomly growing in grid pattern and plane groups. In cell proliferation experiment, cells had a proliferation trend in all composite membrane groups (Fig. 2C).

### Effects of patterns on cellular gene/protein expression

To further assess the effect of patterns on gene expression and protein secretion, a panel of factors was determined by quantitative rt-PCR and western blotting. The detected factors included angiogenesis-related Angpt1, Angpt2 and VEGFA, as well as α-SMA that can promote contraction and wound healing.

**Figure 3.**
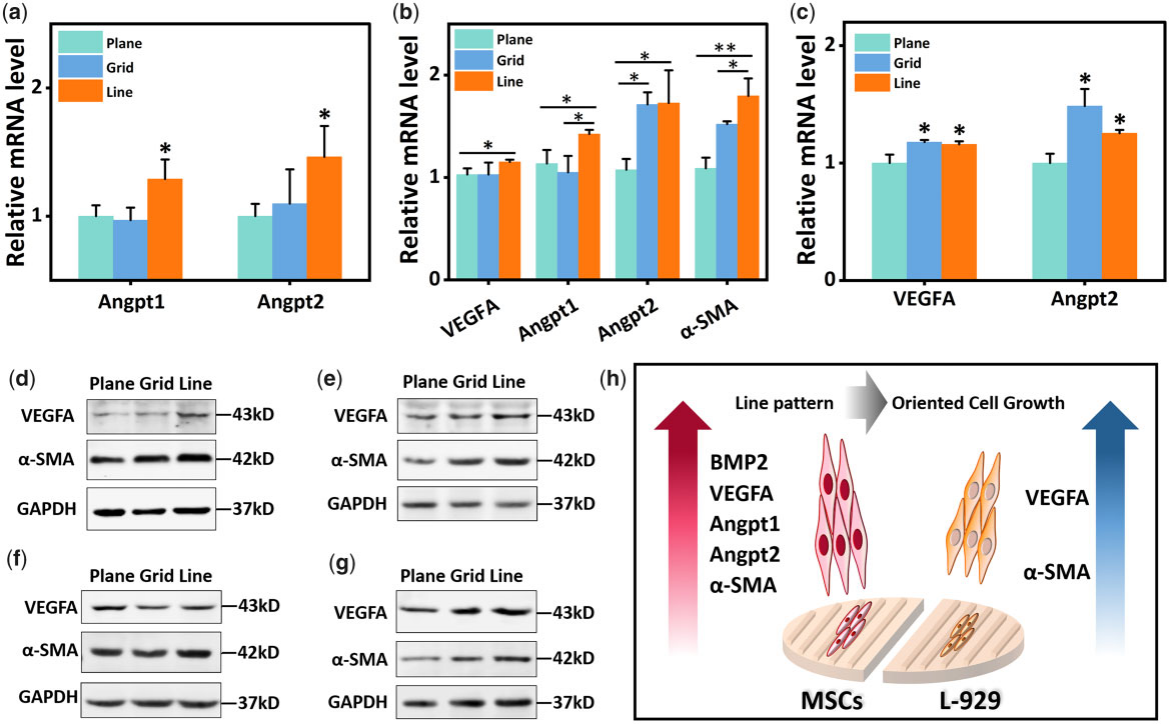
Western blot and rt-PCR analysis results of MSCs and L-929 cells. (**a** and **b**) rt-PCR analysis results of MSCs cultured for 1 and 3 days; (**c**) rt-PCR analysis results of L-929 cells cultured for 3 days; (**d** and **e**) immunoblotting results of MSCs cultured for 1 and 3 days; (**f** and **g**) immunoblotting results of L-929 cells cultured for 1 and 3 days; (**h**) schematic representation of protein expression changes in L-929 cells and MSCs cultured on line patterned membranes. *P < 0.05, **P < 0.01.

For MSCs, after cultured for 1 day, the mRNA levels of Angpt1 and Angpt2 in the line group were significantly higher than the control group (Fig. 3A). After 3 days of culture, in addition to the above three genes, the mRNA expression levels of VEGFA (*P* < 0.05) and α-SMA (*P* < 0.01) in the line group were also significantly higher than the control group (Fig. 3B). At the protein level, the western blot results showed that the expression levels of VEGFA and α-SMA in the line group were higher than those in the control group after 1 and 3 days of culture (Fig. 3D and E).

For L-929 cells, the western blot and rt-PCR results showed no significant difference between the line-patterned composite membranes group and other groups on Day 1. Until the third day of culture, the expression levels of VEGFA and Angpt2 in the grid group and line group were higher than those in the control group at the mRNA level, but there was no significant difference between grid group and line group (*P* < 0.05) (Fig. 3C). However, at the protein level, the expression levels of VEGFA and α-SMA of the line group were higher than the control group and grid group on the third day of culture (Fig. 3F and G). In general, based on the results of rt-PCR and western blot, grid group and line group could promote L-929 cells to secrete VEGFA and α-SMA, and line group had a better effect.

The rt-PCR and western blotting results showed that the line pattern significantly affected L-929 cells and MSCs (Fig. 3H). For L-929 cells, cells cultured in the line pattern would produce more VEGFA and α-SMA, implying the potential to promote neovascularization and wound contraction in the wound area, which could promote wound healing. For MSCs, culturing in line pattern stimulated the cells to secrete more Angpt1, Angpt2 and VEGFA, indicating the potential to promote angiogenesis.

### *In vivo* experiments and histological analysis

**Figure 4.**
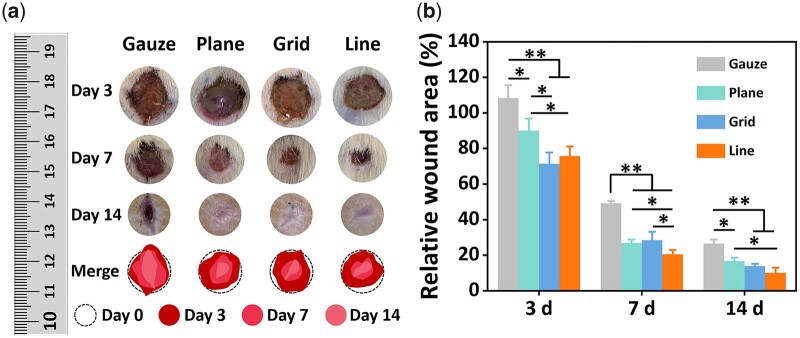
(**a**) Wound images and (**b**) relative wound area on the 3rd, 7th and 14th day after operation. *P < 0.05, **P < 0.01.

In this study, a full-thickness skin defect model with a diameter of 13 mm was used to evaluate the skin repairing ability of the patterned gelatin–PCL/SF composite membrane. From the relative area ratio data of wound healing, gelatin–PCL/SF composite membrane could significantly promote defect healing in rats. The wound areas of the patterned composite membrane groups were significantly smaller than gauze control group at each time point (*P* < 0.01, Fig. 4). On the third day after the operation, the wound area in gauze group was even slightly larger than the original wound area, which may be due to the severe inflammatory response. During the wound healing process, the line pattern group was statistically different from the non-pattern group (*P* < 0.05).

**Figure 5.**
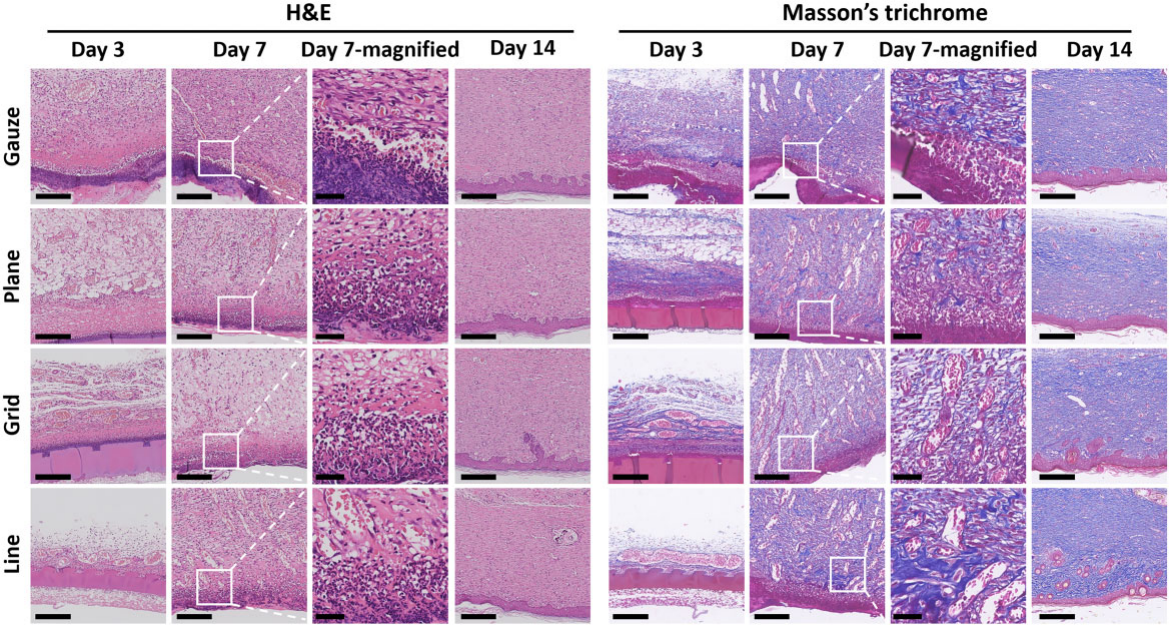
Images of H&E and Masson’s trichrome staining (scale bars: images in Days 3, 7 and 14 columns, 250 μm; images in Day 7-magnified column, 50 μm).

The results of H&E staining showed that the paraffin gauze control group had a more severe inflammatory reaction on the third day, which continued to the seventh day (Fig. 5). Inflammatory response is necessary for wound healing. In the early stage of wound healing, inflammatory cells will phagocytose and clear wound necrotic tissue and bacteria, providing a suitable environment for wound healing. However, prolonged inflammatory response also damages normal tissues, which is not conducive to forming new tissues and hinders skin healing. The three gelatin–PCL/SF composite membrane groups with different patterns had a slight inflammatory response on the third day, and the inflammatory cells were greatly reduced on the seventh day, indicating that the tissue was in a good healing state.

From the Masson’s staining result in Fig. 5, a large number of collagen fibers and some muscle fibers were produced in the four groups. On the seventh day, collagen fibers in composite membrane groups, especially the line pattern composite membrane, were stained darker than in the gauze group, indicating that the composite membrane could promote collagen fiber production. Collagen fibers are abundant in the skin tissue, which can recruit fibroblasts to the wound site, support the new granulation tissue and enhance the toughness. Also on the seventh day, certain amounts of muscle fibers were produced in the new tissue at the wound. When fibroblasts differentiate into myofibroblasts, α-SMA will be secreted, further promoting wound contraction [16].

CK10 is a cytokeratin, which is a late marker for forming epithelial cells in tissues [17]. Epithelial cells are located in the upper layer of the epidermal tissue of the skin, blocking external dust and bacteria, and protecting human tissues [18]. At 14 days after surgery, epithelial tissue was formed in all four groups. The epithelial tissue in the gauze group was thicker, and the line pattern composite membrane group was closest to normal skin tissue (Supplementary Fig. S2).

**Figure 6.**
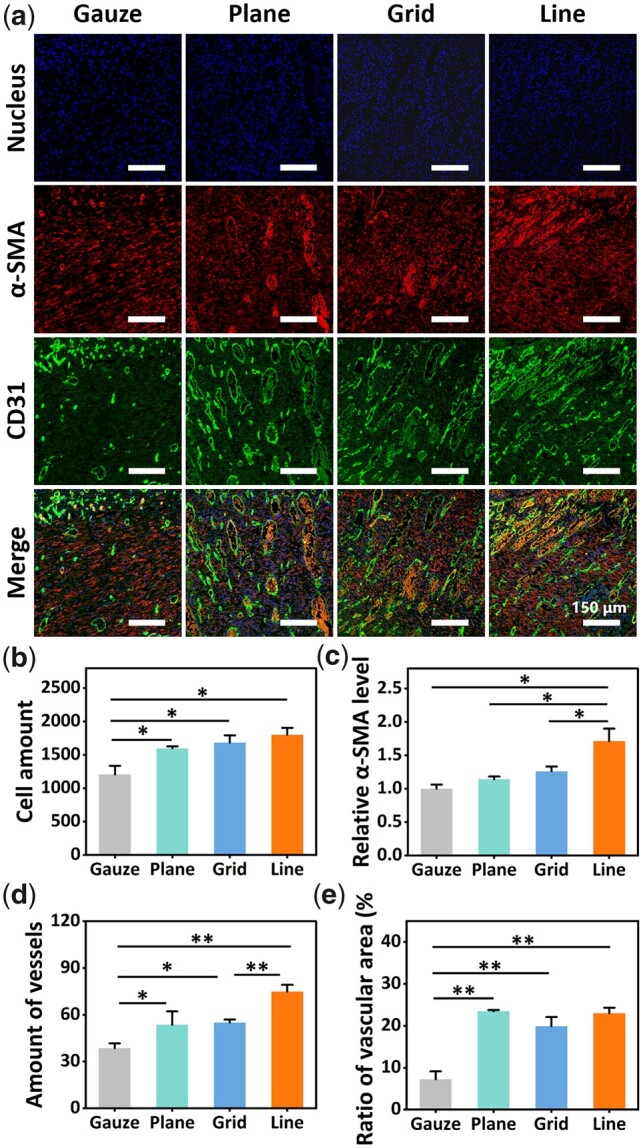
(**a**) Images of nucleus, α-SMA and CD31 in new tissue on the seventh postoperative day. (**b**) Cell amount in visual field; (**c**) ratio of α-SMA fluorescence intensities; (**d**) amount of blood vessels in visual field; (**e**) cross-sectional area of blood vessel in visual field (%) (* indicates that there was a significant difference between this group and the gauze group, *P* < 0.05; **indicates that there was a very significant difference between this group and the gauze group, *P* < 0.01).

From the images of immunostaining above, α-SMA was secreted in all four groups (Fig. 6A), which had a positive effect on promoting skin contraction and accelerating the reduction of the wound area [16]. The semi-quantitative analysis results showed that the expression of α-SMA in line pattern gelatin composite membrane group was the highest and significantly higher than that in other groups (*P* < 0.05) (Fig. 6C). The results indicated that the line pattern gelatin composite membrane might increase the production of α-SMA during wound healing, which was beneficial to promote skin contraction and accelerate wound healing.

CD31 is often used to label vascular endothelial cells, and it is one of the essential indicators for evaluating angiogenesis [19]. Compared with the gauze control group, the wound tissue of the gelatin–PCL/SF composite membrane group had more significant neovascularization on the seventh day, and the number of blood vessels and the proportion of blood vessel cross-sectional area were significantly higher than those of the gauze group (*P* < 0.05). The line pattern composite membrane had relatively more neovascularization (*P* < 0.01) (Fig. 6A, D and E). The formation of new blood vessels provides nutrients and blood oxygen conditions for the proliferation of granulation tissue, which is an indispensable and important condition for wound healing [20]. In addition, the number of cells in all gelatin composite membrane groups was higher than gauze group by cell counting through Image J, and the number of cells in line pattern composite membrane group was the highest (Fig. 6A and B). This indicated that in the middle stage of healing, the gelatin composite membrane might promote cell proliferation and accelerate wound healing. A possible explanation was that the gelatin composite membrane could reduce the inflammatory response in the wound area, while the long-term inflammatory response would damage normal tissue and hinder the generation of new tissue. Therefore, in the new tissue of the wound, the gelatin composite membrane group, especially the line pattern group, had more cells than the gauze group.

## Discussion

The healing process of skin wounds consists of five stages: hemostasis, inflammation, migration, proliferation and maturation [21]. The damaged skin tissue rebuilds under the combined effects of various cells and extracellular matrix (ECM) [22, 23]. To better guide oriented cell growth and promote wound contraction and angiogenesis, we developed a composite membrane with specific micropatterns on the surface to promote wound healing. In this study, line-patterned gelatin membrane not only provided a moist environment to the wound, but also guided cell-oriented growth. Through *in vivo* and *in vitro* experiments, we confirmed that the composite membrane had good biocompatibility and changed the growth direction of cells, promoting cell proliferation and angiogenesis, thereby accelerating wound healing. Also, the line pattern composite membrane could increase the secretion of α-SMA to a certain extent, thereby promoting wound contraction and wound healing.

Among the existing studies, the research on the influence of ECM on cell behavior has been relatively mature. Numerous studies have shown the physical properties of ECM provide mechanical signals to cells that alter the cell’s stretched state by affecting the distribution of F-actin. Mechanical signals are transmitted to the nucleus through the cytoplasm, further activating related signaling pathways and signaling molecules, ultimately changing cell fate. Currently, research on patterned biomaterials is mostly conducted in *in vitro* experiments. There are relatively few *in vivo* experiments about patterned biomaterials, which may be due to the difficulty of controlling the conditions. It is affected by complex *in vivo* environments, material topology maintenance time and other factors. In the present study, patterned composite membranes were used to repair full-thickness skin defects in rats, resulting in promising repair effects with faster healing speed and increased neovascularization. However, more aspects are worth further study, including the mechanism of topological effects on cell behavior, which leads the research more in-depth. Meanwhile, since degradable materials were used in this experiment, the pattern mainly effected on the early stage of wound healing. Non-degradable patterned skin dressings can be used to study the effect of topology at various stages of wound healing. Interestingly, soft materials are more suitable as skin dressings, but materials with a harder surface are more conducive to cell recruitment, adhesion and stretching [24, 25]. How to balance the softness and hardness of the dressing also deserves further research.

## Conclusion

In conclusion, this study developed a patterned biomaterial for skin repair using photolithographic techniques. Due to its plasticity and good biocompatibility, gelatin was used to construct patterned membranes. PCL and SF electrospun membranes were attached to the unpatterned surface of gelatin membranes to improve their mechanical properties. *In vitro* experiments showed that the composite membrane had a pattern that mimicked natural skin, which could guide cells to grow in a direction along the pattern and promote cells to secrete α-SMA and VEGFA, thereby promoting wound contraction and angiogenesis. In addition, the composite membrane had good cell compatibility without affecting cell proliferation. Through *in vivo* experiments, it was confirmed that the composite membrane promoted cell proliferation and angiogenesis, and increased the secretion of α-SMA, thus accelerating wound healing, which was consistent with the results of *in vitro* experiments. This patterned design of biomaterials illustrates the importance of ECM for regulating cell behavior and the possibility of applying topological structures to tissue engineering. It has been widely confirmed that ECM topology can regulate cell fate. Its mechanisms are of great research value and will provide more structural design ideas in biomaterials.

## Supplementary Material

rbac108_Supplementary_Data
