# Supplementary material for: Micropatterned composite membrane guides oriented cell growth and vascularization for accelerating wound healing
Source: Regen Biomater. 2022 Dec 26;10:rbac108. doi: 10.1093/rb/rbac108 (PMC9847515; doi:10.1093/rb/rbac108)
Supplement: rbac108_Supplementary_Data [file rbac108_supplementary_data.docx]

**Micropatterned Composite Membrane Guides Oriented Cell Growth and Vascularization for accelerating wound healing**

Jiaqi Li ^a, b, c, d, 1^, Xulong Liu ^a, b, c, d, 1^, Weiyong Tao ^a, b, c, d^, Yan Li ^a, b, c, d^, Yingying Du ^a, b, c, d, *^, Shengmin Zhang ^a, b, c, d, **^

^a^ Advanced Biomaterials and Tissue Engineering Center, Huazhong University of Science and Technology, Wuhan, 430074, China

^b^ NMPA Research Base of Regulatory Science for Medical Devices & Institute of Regulatory Science for Medical Devices, Huazhong University of Science and Technology, Wuhan 430074, China

^c^ Institute of Biomaterials and Medical Devices, Wuhan Institute of Industrial Innovation and Development, Wuhan 430074, China

^d^ Department of Biomedical Engineering, Huazhong University of Science and Technology, Wuhan 430074, China

* Corresponding author: Advanced Biomaterials and Tissue Engineering Center, Huazhong University of Science and Technology, Wuhan, 430074, China

** Corresponding author: Advanced Biomaterials and Tissue Engineering Center, Huazhong University of Science and Technology, Wuhan, 430074, China

E-mail addresses: yingyingdu@hust.edu.cn (Y. Du), smzhang@hust.edu.cn (S. Zhang).

^1^ These authors contributed equally to this work.

**Keywords:** Topology; Cell behavior; Wound healing; Gelatin; Polymer biomaterials

Table S1. Sequences of primers used for gene expression analysis

| Rat Genes | Primer sequences |
| --- | --- |
| Angpt1 | Forward: GAAGGGAACCGAGCCTACTC |
|  | Reverse: GGGCACATTTGCACATACAG |
| Angpt2 | Forward: TCCAGACTGACGCACATCAC |
|  | Reverse: ATTTCTCCAGACCCGCAGTG |
| VEGFA | Forward: AAAGCCAGCACATAGGAGAG |
|  | Reverse: AGGATTTAAACCGGGATTTC |
| α-SMA | Forward: AGGGAGTGATGGTTGGAATG |
|  | Reverse: GATGATGCCGTGTTCTATCG |
| GAPDH | Forward: CTGGAGAAACCTGCCAAGTATG |
|  | Reverse: GGTGGAAGAATGGGAGTTGCT |
|  |  |
| Mouse Genes | Primer sequences |
| Angpt2 | Forward: ACGGTCAACAACTCGCTCCT |
|  | Reverse: TTCCGCACAGTCTCTGAAGGT |
| VEGFA | Forward: CTTGTTCAGAGCGGAGAAAGC |
|  | Reverse: ACATCTGCAAGTACGTTCGTT |
| GAPDH | Forward: CATGTTCCAGTATGACTCCACTC |
|  | Reverse: GGCCTCACCCCATTTGATGT |

**
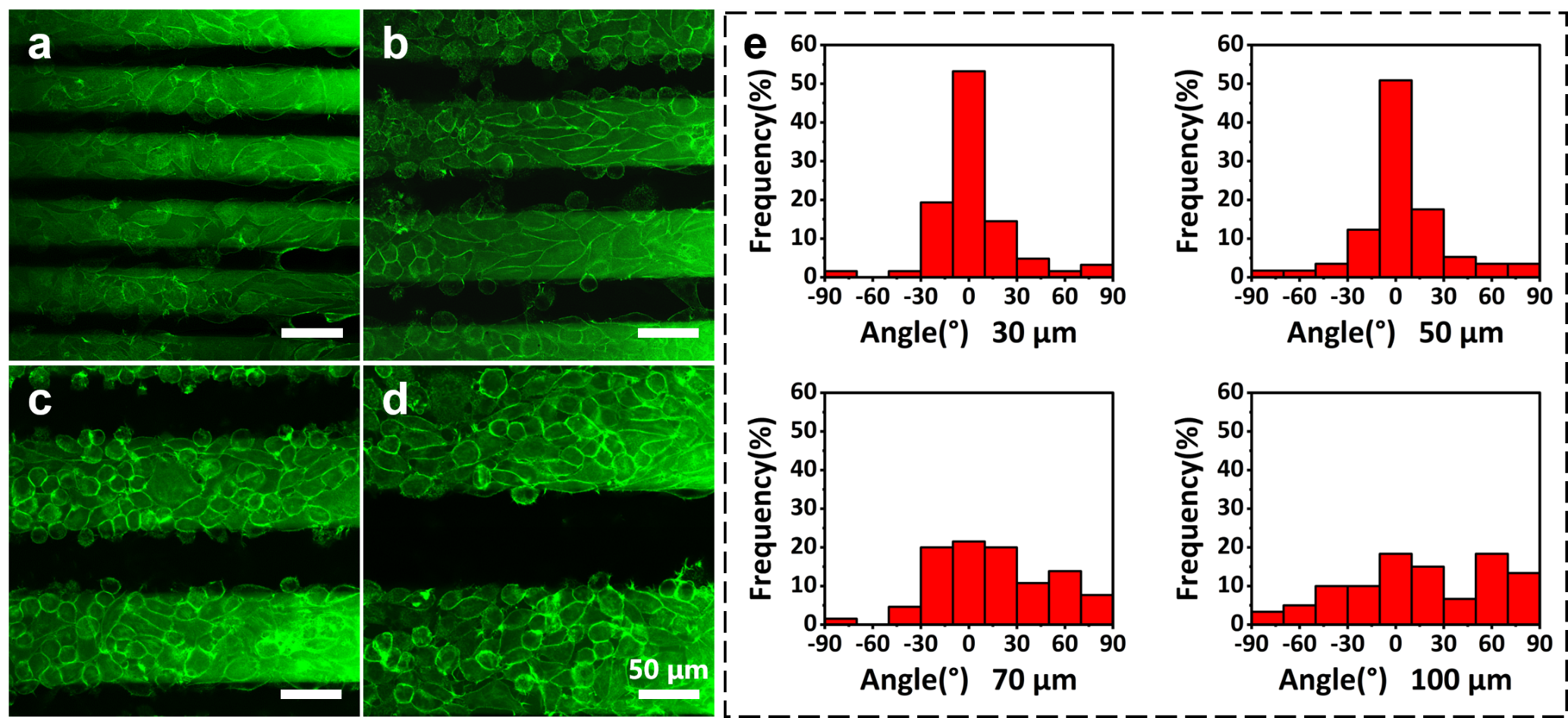
**

Fig. S1. Confocal images of L-929 cells on line-patterned gelatin membranes at different intervals: (a) 30 μm; (b) 50 μm; (c) 70 μm; (d) 100 μm. (e) Statistics of the stretching direction of L-929 cells on different sizes of line pattern gelatin membranes.


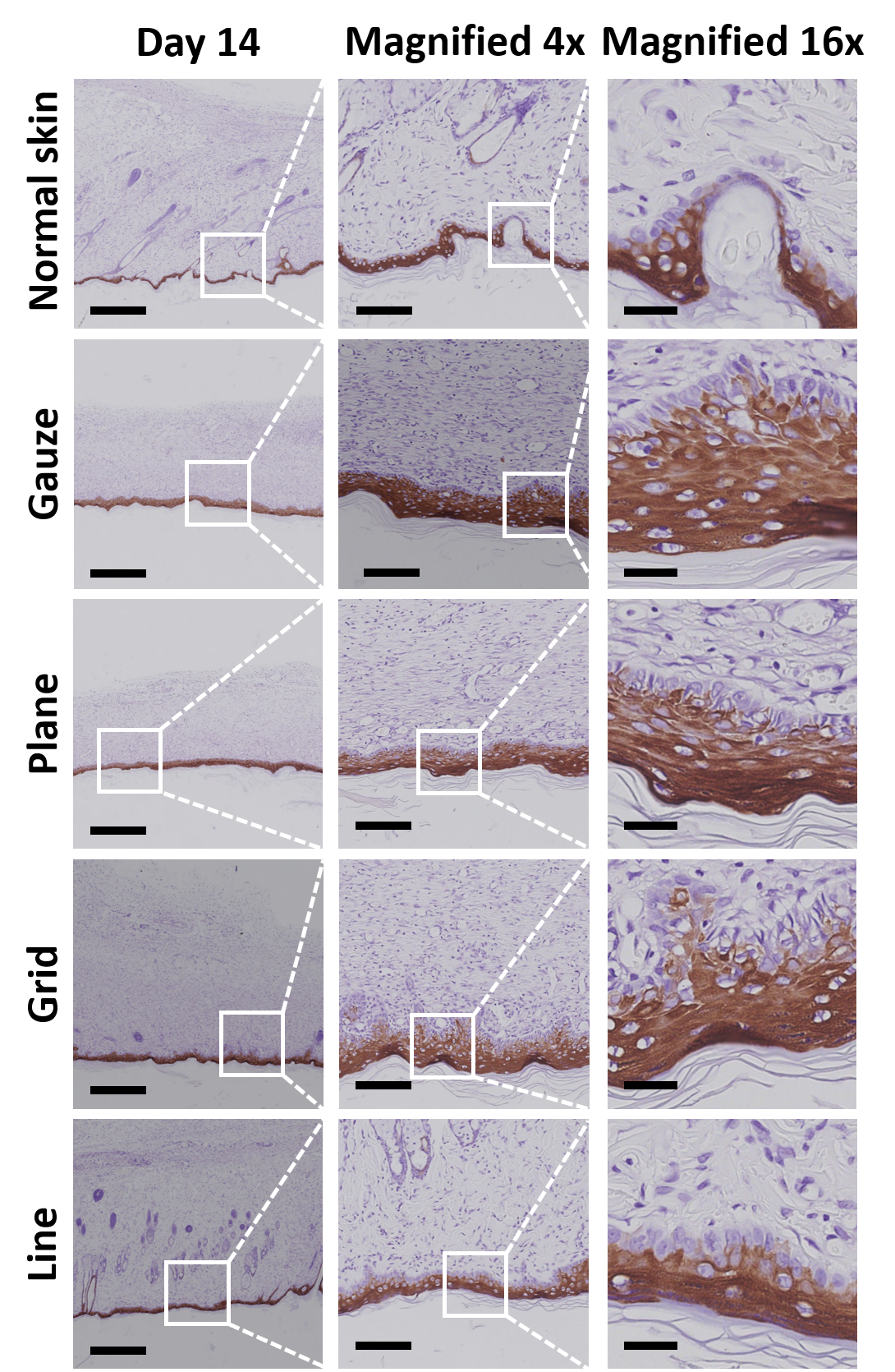


Fig. S2. CK10 immunohistochemical staining images of new tissue on the 14th postoperative day (Scale bars: images in Day 14 columns, 500 μm; images in Magnified 4x columns, 125 μm; images in Magnified 16x columns, 30 μm).
